# Ignoring the Impact of Fermentation Could Result in Substantial Misestimation of Folate and Cobalamin Adequacy: A Simulation Study on Injera Consumption in the Ethiopian Context

**DOI:** 10.1016/j.cdnut.2025.104581

**Published:** 2025-03-03

**Authors:** Eric O Verger, Sonia Fortin, Aynadis Tamene, Henok Ashagrie, Claire Mouquet-Rivier, Christèle Humblot

**Affiliations:** 1 MoISA, Univ Montpellier, CIRAD, CIHEAM-IAMM, INRAE, IRD, L’Institut Agro, Montpellier, France; 2 Qualisud, Univ Montpellier, IRD, Institut Agro, CIRAD, Avignon Université, Univ de La Réunion, Montpellier, France; 3 Center for Food Science and Nutrition, College of Natural and Computational Sciences, Addis Ababa University, Addis Ababa, Ethiopia

**Keywords:** vitamin B9, vitamin B12, nutrient adequacy, fermented food, resource-poor settings

## Abstract

B-vitamin content of plant-based foods can be deeply modified by fermentation, particularly the active cobalamin form, which is often considered to be zero in food composition databases. We simulated the consequences of including or excluding the impact of fermentation in estimating folate and cobalamin adequacy using secondary data obtained from a survey of 323 women in Ethiopia plus the vitamin content of *injera* (fermented flat bread) reported in the literature. As folate content can change during fermentation, the prevalence of inadequacy in scenarios that include the effect of fermentation was higher (90%) or lower (67%) than in the original data. Our simulation based on data obtained using cobalamin-producing microorganisms lowered the prevalence of inadequacy to 54%. Ignoring the impact of fermentation may result in substantial misestimation of folate and cobalamin adequacy in Ethiopia, and it should be evaluated in other contexts in which fermented foods are consumed as staple foods.

## Introduction

B-vitamins such as folate (vitamin B9) and cobalamin (vitamin B12) are water-soluble vitamins playing key roles in human metabolism. They function as cofactors, coenzymes, and transcription control factors. Because humans lack the ability to synthesize these vitamins, they must be obtained from dietary sources. Unfortunately, folate and cobalamin deficiencies are prevalent in many countries, particularly in low-income regions [[1], [2], [3]]. These deficiencies pose a severe threat to human health, leading to devastating and life-threatening conditions and contributing to anemia burden, especially in vulnerable groups such as children and pregnant women [4].

Fermented foods are staple foods in many countries, and fermentation can strongly influence the folate and cobalamin content of the raw material due to the action of microorganisms that either consume the folate that was originally present or produce folate and/or cobalamin [[5], [6], [7]]. Thus, spontaneous and backslopping fermentation produce final products with highly variable folate content, and estimations of its contribution to vitamin requirements based on the raw material are not accurate. In international guidelines and food composition tables [8,9], cobalamin values have often been assumed to be zero in unfortified plant-based foods, with the exception of fermented foods. However, a pioneer work on *tempeh* (produced from soy) showed that significant amounts of cobalamin were present [7]. However, overall, analyses of cobalamin concentrations are rarely conducted on fermented products [10,11]. As a result, cobalamin values are likely to be considered zero by default, even in fermented products of plant origin, which, in turn, may lead to errors in estimating the prevalence of inadequate intakes in contexts where fermented products are consumed as staple foods. The bioavailability of folate from food is typically estimated to be ∼50% when nutritional recommendations are formulated [4]. However, results in the literature concerning the bioavailability of natural folates compared with that of folic acid are not always in agreement and range from 44% to 80% of that of folic acid [12]. In contrast, cobalamin is only synthesized by certain microorganisms, which are also able to produce different cobalamin analogs (corrinoids), not biologically active in humans [13].

*Injera* is a fermented, thin, soft, round, sour-tasting flatbread, often prepared from teff, a widely consumed staple in Ethiopia [14]. Recent work on samples collected in different households in Addis Ababa, showed that fermentation of teff flour during *injera* preparation significantly increased the corrinoid content, leading to a final product with ≤5.7 μg/100 g, i.e., theoretically fully able to meet the recommended nutritional intake because *injera* consumption is between 130 and 202 g/d [6]. Although the corrinoid identified was the biologically inactive form [15], these results are promising because the efficiency of using bacteria selected for their ability to produce the active cobalamin form has been demonstrated at the laboratory scale [15]. Furthermore, it was shown that depending on the microorganisms responsible for this so-called “natural fermentation,” the folate content in *injera* can increase or decrease during fermentation [5,6]. The use of folate-producing bacteria isolated from *injera* dough was also shown to efficiently maximize folate production [15].

The aim of the present study was thus to measure the difference between estimates of folate and cobalamin intake adequacy depending on whether or not the impact of fermentation during *injera* preparation is taken into consideration. To this end, secondary quantitative 24-h recall data from a survey of 323 women in Ethiopia were used along with folate and cobalamin contents reported in the literature. Data on samples collected both in the field and from samples prepared in the laboratory from folate and cobalamin-producing microorganisms were used to show maximum potential intake adequacy.

## Methods

### Study population

The original dietary data used for this study were downloaded from the FAO/WHO GIFT open-access online platform (https://www.fao.org/gift-individual-food-consumption/en). The study protocol is described in detail elsewhere [16]. Briefly, the study was conducted in a rural community located in the district of Butajira, Southern Ethiopia, from the 2 July to 30 August 2013. Simple random sampling was used to recruit 159 pregnant women and 164 women who were not pregnant (mean age of the whole sample was 29.8 y). Dietary intake data were collected using multiple-pass interactive 24-h recall interviews with the women in their own homes. Portion sizes were estimated using common household measures (e.g., a spoon). A second 24-h recall was repeated with 41 pregnant women and 25 women who were not pregnant belonging to the same sample and used to adjust for day-to-day variations in nutrient intakes. The Ethiopian food composition table was used to calculate nutrient content [17].

### Nutritional composition simulation

In the dietary survey used for this study, folate content was reported but not cobalamin content. We consequently used the cobalamin content for animal products listed in the 2018 Kenyan food composition table [18] while assuming zero cobalamin content for all plant-based human foods, including *injera*. In the simulation, we assumed that the folate and cobalamin contents from the dietary survey were similar irrespective of the type of teff (red or white) or the proportion of teff flour to that of the other flours (maize or millet) used in the preparation of the *injera* (from 25% to 100%), whereas in the original dietary data, according to these criteria, the folate content of *injera* varied from 0 to 18 μg/100 g fresh base.

Next, we simulated the effect of including the impact of fermenting teff batter during the preparation of *injera* on folate and cobalamin contents. In 3 scenarios (Sc1, Sc2, and Sc3), we replaced the folate content of *injera* with minimum, mean, and maximum values (7.4, 15.1, and 31.7 μg/100 g food folate fresh basis, respectively) measured on samples of teff-based *injera* taken from 10 to 20 randomly selected households in the 10 subcities of Addis Ababa [5,6]. In another scenario (Sc4), we included data on teff-based *injera* prepared in the laboratory using 1 folate-producing bacterium (31.4 μg/100 g fresh basis [15]). The measurements were taken using a microbiological assay [19]. In the final scenario (Sc5), we included laboratory data obtained using 1 cobalamin-producing bacterium (2.5 μg/100 g fresh basis [15]). The measurements were taken using the microbiological assay kit (P1002, r-Biopharm) and a 96-well microwell plate.

### Estimation of usual intakes

The usual intake distribution of folate and cobalamin was estimated using the multiple source method (MSM) online interface (https://msm.dife.de/, accessed on the 6 May 2024). Briefly, the MSM is a linear regression model in which the predicted consumption and the corresponding model residuals are estimated with covariates, and the residuals of the linear regression model are then transformed to normality by a 2-parameter BOX-COX transformation [20]. Due to the absence of food frequency data, we used the MSM default option, assuming all participants are habitual consumers of folate and cobalamin. Covariates in the usual intake models included age (years) and pregnancy status.

### Statistical analyses

The average dietary pattern of the participants was described using elemental statistics (mean, SD) of consumption of the 15 food groups listed on the Minimum Dietary Diversity for Women classification (10 main food groups plus 5 additional food groups, [21]). To assess the adequacy of the nutrient intakes, we used the harmonized estimated average requirement (EAR) values for nonpregnant and pregnant women: 250 μg and 520 μg of folate for nonpregnant and pregnant women, respectively, and 2.0 μg and 2.2 μg of cobalamin for nonpregnant and pregnant women, respectively [22]. Based on the MSM estimates of usual folate and cobalamin intakes, we calculated the prevalence of inadequate intakes using the EAR cut-point method, as well as the probabilities of adequacy, which combine the distributions of requirements and intakes within the group to estimate the expected proportion of individuals at risk of inadequacy. The EAR cut-point method is simpler than the probability approach, because it only counts how many individuals in the group of interest have usual intakes that are below the EAR [23].

## Results

### Description of the dietary pattern

**FIGURE 1:**
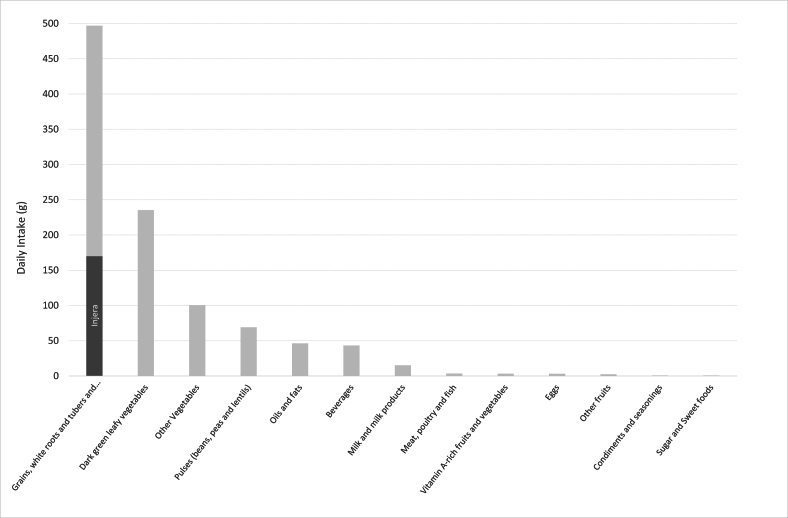
Average dietary patterns of nonpregnant and pregnant women living in Butajira district, Southern Ethiopia (*n*  = 313).

Figure 1 shows the average food group intakes calculated directly from the observed values. The dietary pattern was characterized by a plant-based diet, mainly composed of grains, white roots and tubers and plantain (mean 497 g/d), dark green leafy vegetables (235 g/d), other vegetables (100 g/d), and pulses (69 g/d). Specifically, the average consumption of *injera* was 170 g/d among the total sample and 442 g/d among the consumers only (38% of the total sample). Consumption of animal source foods was extremely low, with 15.2 g/d of milk and milk products and 3.5 g/d of meat, poultry, and fish. There was no difference in consumption between nonpregnant and pregnant women, except for 3 food groups that are not widely consumed: milk and milk products, eggs, and other fruits (data not shown).

### Usual intakes and nutrient adequacy

Table 1 lists the usual intakes, probabilities of adequacy, and prevalence of inadequacy across the original data and the simulations. In the original data, the usual intake of folate was 226 ± 77 and 208 ± 61 μg/d in pregnant and nonpregnant women, respectively, and the usual intake of cobalamin was 0.29 ± 0.63 and 0.09 ± 0.43 μg/d, respectively. The probabilities of adequacy were low (<0.17), and prevalence of inadequacy was high (>87%) for both nutrients and the 2 groups of women.

**TABLE 1:** Usual intakes, probability of adequacy, and prevalence of inadequacy across the original data and the simulations in nonpregnant and pregnant women living in Butajira district, Southern Ethiopia (n  =  313).

Nutrient	Scenario	Content er 100 g in*injera* (μg)	Nonpregnant women (*n* = 164)			Pregnant women (*n* = 159)		
			UsualIntake^1^	Probabilityof adequacy^1^	Prevalence ofinadequacy (%)	UsualIntake^1^	Probabilityof adequacy^1^	Prevalence ofinadequacy (%)
Folate	Original data	(0–18)	208 (61.0)	0.17 (0.29)	86.6	226 (77.3)	0.01 (0.08)	99.4
	Sc1	7.4	199 (29.0)	0.15 (0.25)	90.2	213 (75.0)	0.01 (0.06)	99.4
	Sc2	15.1	211 (45.9)	0.16 (0.26)	88.4	226 (81.8)	0.02 (0.10)	98.7
	Sc3	31.7	240 (99.1)	0.35 (0.40)	67.1	256 (103)	0.04 (0.16)	96.9
	Sc4	31.4	239 (98.7)	0.35 (0.40)	67.1	255 (103)	0.04 (0.16)	96.9
Cobalamin	Original data	0	0.09 (0.43)	0.01 (0.11)	98.8	0.29 (0.63)	0.03 (0.15)	97.5
	Sc5	2.5	4.20 (6.31)	0.36 (0.48)	64.0	4.70 (4.68)	0.46 (0.50)	54.1

In the original dietary data, the folate content of *injera* ranged from 0–18 μg/100 g fresh basis according to the type of teff (red or white) and the proportion of teff flour to that of the other flours (maize or millet) used in the preparation (25%, 50%, 75%, or 100%). Sc1: folate content of *injera* with minimum value measured in a sample of teff-based *injera* (7.4 μg/100 g food folate fresh basis); Sc2: folate content of *injera* with average value measured in a sample of teff-based *injera* (15.1 μg/100 g fresh basis); Sc3: folate content of *injera* with maximum value measured in a sample of teff-based *injera* (31.7 μg/100 g fresh basis); Sc4: folate content of teff-based *injera* prepared at laboratory scale using one folate-producing bacterium (31.4 μ/100 g fresh basis); Sc5: cobalamin content of teff-based *injera* prepared at laboratory scale using one cobalamin-producing bacterium (2.5 μg/100 g fresh basis).

1 Values are means (SD).

Using the minimum and average values of folate measured in samples of teff-based *injera* (Sc1 and Sc2) barely changed the usual intakes and nutrient adequacy. Using the maximum values of folate measured in samples collected in Ethiopia and values measured in *injera* prepared in the laboratory (Sc3 and Sc4), in both scenarios, the usual intake of folate by nonpregnant and pregnant women was higher than revealed by the original data. As a result, the prevalence of inadequacy was divided by 1.29 in nonpregnant women compared with the original data, whereas the prevalence of inadequacy barely changed in pregnant women.

When a cobalamin-producing bacterium was used in the laboratory (Sc5), we observed a dramatic increase in the usual intake of cobalamin from 0.09 μg to 4.20 μg in nonpregnant women and from 0.29 to 4.70 μg in pregnant women, i.e., the prevalence of inadequacy was divided by 1.54 and 1.80 in nonpregnant and pregnant women, respectively, compared with the original data.

## Discussion

In this simulation study based on dietary data collected in a survey of 323 women in Ethiopia, we observed that, depending on whether the impact of fermentation during *injera* preparation is taken into consideration or not, we could go from having substantially similar results to major differences between estimates of folate and cobalamin adequacy. These differences in estimates could have been even greater if the prevalence of *injera* use had been higher in the original data. Because ∼80% of the sample had a single 24-h dietary recall, we may well have underestimated the prevalence of true *injera* consumers in the survey area (38% in the study). Moreover, *injera* consumption is higher in urban areas and even higher in big cities than in rural areas like the one in which the original data were collected [24].

Given that the folate content of *injera* can be lower, similar, or higher than that of the raw material (teff), the prevalence of inadequacy in the scenario that included the effect of fermentation could be higher (90%), similar (88%), or lower (67%) for nonpregnant women than in the original data. Because the requirement for pregnant women is higher, the prevalence of inadequacy only decreased slightly when higher folate contents were used. Another study conducted in different parts of Ethiopia showed that the proportion of people with low folate intake is basically the same as the proportion of people with low serum folate [3]. Fermentation using folate-producing microorganisms isolated from different fermented cereal-based foods has been proven to be effective in increasing their folate content and can cover all or part of the daily requirements of women and children [15,25]. The use of such a strategy would be a way to maximize the folate content of *injera* by limiting the consumption of folate by endogenous microorganisms during fermentation.

In our simulation, the high prevalence of inadequacy for cobalamin was lowered to 54% in pregnant women. Because the corrinoid detected in large quantities in samples of *injera* collected in Ethiopia was inactive for humans, we based our simulation on data obtained in the laboratory using a cobalamin-producing bacterium [15]. As discussed by the authors of that study, different microorganisms involved in injera fermentation, such as members of the *Propionibacterium freudenreichii* species, are known for their cobalamin-producing properties [5]. As only a few samples were analyzed, it is possible that other samples of *injera* contain the active form. A recent study conducted in Ethiopia showed that despite the fact that 100% of the adult population had inadequate dietary intakes of vitamin B12, low serum cobalamin was detected only in 23%–25% of the population [3]. The hypothesis of the role of fermentation in the production of cobalamin was not raised by the authors. Recent studies demonstrated the feasibility of meeting the recommended daily intake of the active form of cobalamin with only 100 g/d of bread fortified by fermentation [26]. Similarly, *injera* has been successfully fortified with the active form of cobalamin, thereby theoretically enabling full coverage of cobalamin requirements [15].

Certain limitations need to be considered when interpreting the results of our study. Using a method other than the MSM to estimate the usual intake distributions of nutrients could have produced different results, although the MSM has been shown to produce estimates that are as good as those obtained using other methods [27]. In addition, having more participants with repeated 24-h recall would modify the day-to-day variation in nutrient intakes and, hence, the estimates of usual intake distributions. Similarly, data on episodically consumed foods would have improved our estimates of the distribution of habitual intakes. A further limitation is that the dietary survey was conducted at a single location over a 2-mo period [16], meaning our results cannot be generalized.

In conclusion, our study demonstrated that ignoring the impact of fermentation during *injera* preparation may lead to substantial misestimation of folate and cobalamin adequacy in the Ethiopian context. More generally, our study illustrates the importance of investing in analysis of micronutrient contents, particularly of cobalamin, and in identifying its active forms in order to avoid major misestimation of the prevalence of inadequate nutritional intakes. This microbial biofortification approach should now be evaluated in intervention trials to estimate the feasibility of this promising alternative approach for overcoming vitamin deficiencies.

## Author contributions

The authors’ responsibilities were as follows – EOV, SF, CM, CH: designed the study; AT, HA: provided folate and cobalamin content values; EOV, SF: analyzed the data; SF: drafted the figures and tables; EOV, CH: wrote the manuscript; SF, AT, HA, CM: critically reviewed; and all authors: read and approved the final manuscript.

## Data availability

Data described in the manuscript are available on the FAO/WHO GIFT online platform (https://www.fao.org/gift-individual-food-consumption/en). The code book and analytic code will be made available upon request, pending application and approval by authors of the current study.

## Funding

The authors reported no funding received for this study.

## Conflicts of interest

The authors report no conflicts of interest.
